# Optimizing Time Resolution Electronics for DMAPs

**DOI:** 10.3390/s23135844

**Published:** 2023-06-23

**Authors:** Enrique López-Morillo, Clara Luján-Martínez, José Hinojo-Montero, Fernando Márquez-Lasso, Francisco Rogelio Palomo, Fernando Muñoz-Chavero

**Affiliations:** Department of Electronic Engineering, University of Sevilla, 41092 Sevilla, Spain; cilujan@us.es (C.L.-M.); jhinojo@us.es (J.H.-M.); fmarquez1@us.es (F.M.-L.); fpalomo@us.es (F.R.P.); fmunoz@us.es (F.M.-C.)

**Keywords:** depleted monolithic active pixel sensors (DMAPSs), timing, time walk, pixel detector, Large Hadron Collider (LHC), low power, area efficiency

## Abstract

Depleted Monolithic Active Pixel Sensors (DMAPSs) are foreseen as an interesting choice for future high-energy physics experiments, mainly because of the reduced fabrication costs. However, they generally offer limited time resolution due to the stringent requirements of area and power consumption imposed by the targeted spatial resolution. This work describes a methodology to optimize the design of time-to-digital converter (TDC)-based timing electronics that takes advantage of the asymmetrical shape of the pulse at the output of the analog front-end (AFE). Following that methodology, a power and area efficient implementation fully compatible with the RD50-MPW3 solution is proposed. Simulation results show that the proposed solution offers a time resolution of 2.08 ns for a range of energies from 1000 e^−^ to 20,000 e^−^, with minimum area and zero quiescent in-pixel power consumption.

## 1. Introduction

High-energy physics (HEP) experiments require high-spatial- and time-resolution pixel detectors. The traditional solution for experiments in the Large Hadron Collider (LHC), such as ATLAS [1], ALICE [2], CMS [3], and LHCb [4], was the use of hybrid pixel detectors. In these detectors, the sensor and the readout electronics are manufactured independently. This enables designers to choose the most appropriate integration technology to optimize the performance of each part. However, it significantly increases both production and assembly costs, making them critical for large detectors with multiple layers and thousands of chips, such as the CMS system.

In recent years, Monolithic Active Pixel Sensors (MAPSs) [5] have gained popularity due to the possibility of integrating the sensing diode and the readout electronics on the same substrate [6], which reduces costs and production time. In this context, Depleted Monolithic Active Pixel Sensors (DMAPSs), built on high-resistivity substrates, are a natural evolution of MAPSs. The use of a high electric field applied to these substrates results in a depleted sensor [7], which operates with increased efficiency in charge collection, higher speed, and higher radiation tolerance. However, unlike hybrid solutions, DMAPSs do not benefit from aggressive technology downscaling because there are no nanometric processes based on a high-voltage and high-resistivity substrate. Moreover, in the case of DMAPSs, limiting the sensor leakage current requires the use of guard rings, and increasing the breakdown voltage of the sensor implies separating the different electrodes. Therefore, for the same pixel size, a smaller active area is available for the readout electronics [8]. This significantly increases design constraints, and, thus, many attempts have been made by the scientific community to develop monolithic solutions that perform high spatial and temporal resolution that current and future HEP experiments require.

High spatial resolution requires a small pixel area, which limits the amount and complexity of electronics dedicated to the time acquisition that can be included in the pixel and, consequently, the time resolution. This also has an impact on the maximum pixel power consumption, as the power density is limited by temperature constraints. Therefore, the natural tendency is to depopulate the pixel and take most of the processing electronics to the periphery, which complicates the routing. This strategy can cause signal integrity issues, especially in a large matrix. Moreover, most of the area occupied by routing is prone to become a non-detection zone, which will degrade the final spatial resolution. Therefore, in DMAPs, there is a trade-off between the spatial resolution, the routing area, and/or the pixel size.

**Figure 1:**
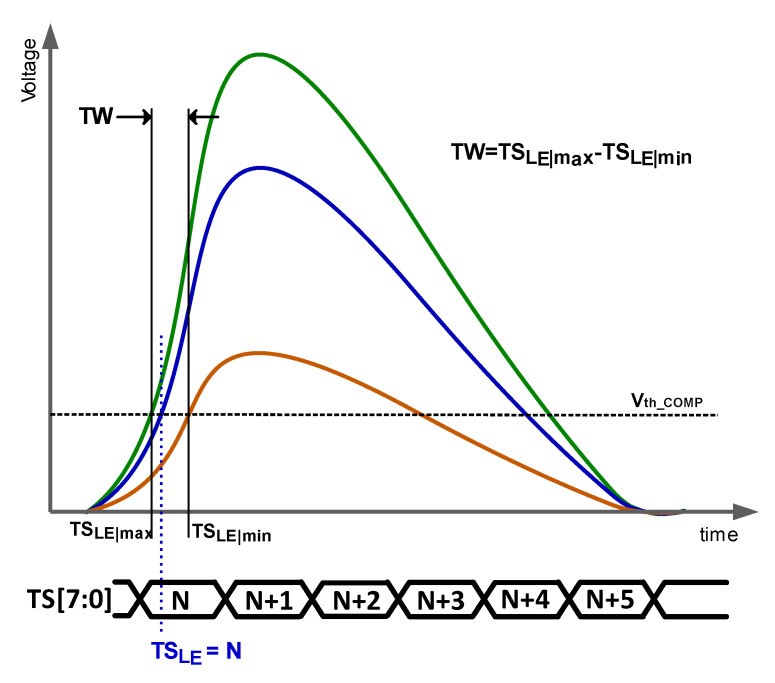
Graphical illustration of the time walk.

Time resolution is determined by the accuracy with which the time of arrival (ToA) is measured. The ToA is defined as the time in which the signal induced by the detected particle exceeds a threshold voltage. As particles that deposit different amounts of energy induce signals of different amplitudes and rise times, different ToAs are expected to be measured for particles with different energies, even when they impact at the same instant. This effect, known as time walk (TW), is responsible for determining the time resolution of the whole system. Note that the ToA can only be acquired with the accuracy of the system timestamp (TS), and, consequently, in the literature, the terminology TS of the leading edge (TS_LE_) is preferred. In practice, accuracies of a few nanoseconds are targeted in HEP. As the sensor reacts in a few hundred picoseconds, the readout electronics is the bottleneck. Figure 1 shows the signal at the output of the charge-sensing amplifier (CSA) for three hits with different amounts of energy. That figure illustrates the definition of TW, which is calculated for a certain energy range as the difference between the stored TS_LE_ of the particles with minimum energy (TS_Lemin_) and maximum energy (TS_Lemax_).

As mentioned above, reducing the TW will have a direct impact on the system time resolution. However, any proposed solution faces stringent requirements because of the high granularity desirable in HEP experiments. This forces the minimization of area and power consumption of the in-pixel electronics and implies limited routing resources at the matrix level due to signal integrity issues and the required area for the interconnections.

This work focuses on optimizing the timing electronics for DMAPs in the context of the RD50 collaboration. Therefore, our solution must be fully compatible—in terms of analog interface and pixel readout strategy—with RD50-MPW3, their current solution.

The rest of the document is as follows: Section 2 describes the state-of-the-art (SoA) techniques to improve the time resolution in DMAPS and highlights the main objectives of this work, Section 3 presents the RD50-MPW3 solution, Section 4 focuses on the timing resolution, providing a theoretical analysis aiming to optimize the required accuracy of the ToT, Section 5 details the proposed architecture, Section 6 will discuss some simulation results, and, finally, in Section 7, some conclusions are drawn, and future work is outlined.

## 2. State of the Art

Several methods to improve the time resolution of detectors have been proposed in the literature.

The straightforward solution is to *reduce the TW by improving the analog interface* and specifically increasing the speed of the amplifier [9]. This equalizes the slope of the amplified signal for different energies but requires higher power and area consumption in the pixel, which has a negative impact on the granularity of the system (1 mm^2^ pixel area). Moreover, in current and future applications where a TW in the order of a few nanoseconds is targeted, the required power and area consumption are untenable.

Other authors include *additional circuitry in the analog interface to compensate for the different delays that the signal suffers depending on its energy, performing an in-pixel correction of the measured ToA*. This is the case, for example, of the time-walk-compensated comparator (TWCC) method [10], which uses two comparators with different threshold voltages. The first comparator triggers a circuit that generates a delay proportional to the amplitude of the output signal of the amplifier, while the second digitalizes the compensated signal and delivers the corrected time stamp. Another example is the two-threshold method [10], which also uses two comparators with different thresholds, but in this case, the first threshold is very low, close to the noise level, ensuring a measurement of the ToA with small TW. To avoid the detection of false hits, the second comparator with a higher threshold voltage confirms the event. MuPix8 has successfully implemented these two methods, achieving a time resolution of 25 ns for an 80 μm × 81 μm pixel in a 128 × 200 matrix for energies between 1000 e^−^ and 10,000 e^−^ [11]. As the discriminator is in the periphery of the matrix—outside of the pixel—the main drawback of this approach is that different delays are obtained in pixels hit with the same energy due to the length of the connection between each pixel and the discriminator [12]. This is an illustrative example of how increasing the routing can limit the effective time resolution.

**Figure 2:**
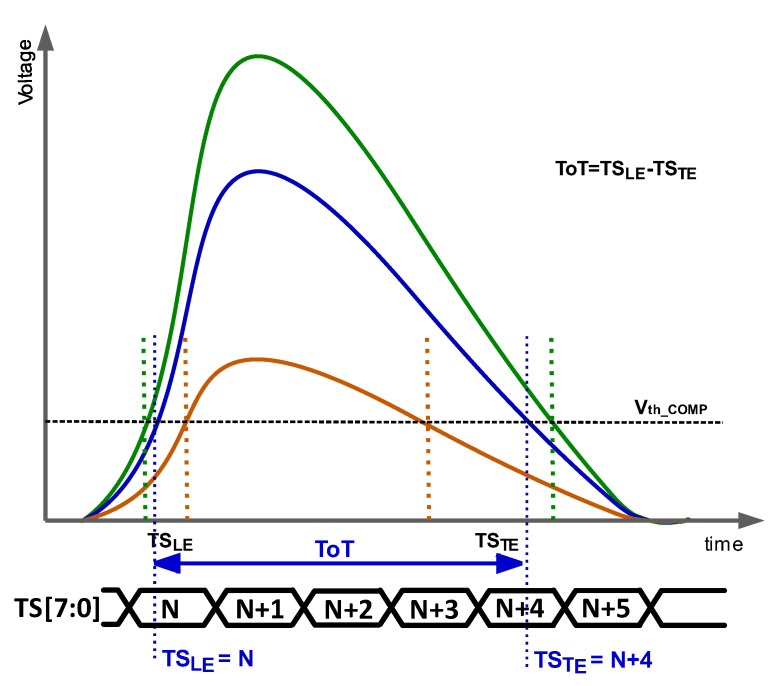
ToT for different energies.

The most common option to reduce the TW is to perform *off-pixel correction using a measurement of the particle energy, usually the signal height or the time over threshold (ToT)*. ToT (Figure 2) is the time during which the amplified signal exceeds the threshold voltage and is defined as

(1) $$\mathrm{ToT}={\mathrm{TS}}_{\mathrm{LE}}-{\mathrm{TS}}_{\mathrm{TE}}$$

where TS_LE_ and TS_TE_ are given by the moments when the amplified signal surpasses and crosses down the discriminator threshold, respectively. As mentioned above, the TS_LE_ is the stored time corresponding to the ToA. From Figure 2, it is established that the larger the ToT, the higher the energy of the particle.

This method alone usually does not offer sufficient time resolution for most applications. Specifically, when the maximum voltage amplitude of the amplified signal is used as an estimation of the energy of the particle, it is necessary to include a high-resolution ADC in every pixel. This ADC is not feasible to integrate within the pixel due to the strict area and power consumption limitation. Therefore, placing it in the periphery, totally or partially shared by the pixels in the matrix, is imperative. The high hit rate of these systems implies the need for a high-speed ADC and, thus, high complexity, large area, and excessive power consumption. Furthermore, the ADC resolution can be compromised by the propagation time of the signals from every pixel of the matrix. Moreover, the distribution of the signal delays according to their energies and pixel position is not linear, forcing the use of a nonlinear ADC, in which the thresholds must be tuned independently for each pixel [10]. The tuning structures will occupy additional space and power consumption. On the other hand, when the ToT is used, the resolution is limited by the TS. Drastically increasing the frequency of the clock complicates the routing in terms of signal integrity.

According to the literature, the solutions that perform better time resolution are those that use *off-pixel correction but implement additional mechanisms inside the pixel to increase the accuracy of the measurement of ToT and/or the energy of the particle.* Therefore, the search for smart solutions to correct the TW adding minimal complexity to the in-pixel electronics and minimizing the required extra lines for the readout of the more accurate measurements is focusing most of the current efforts.

For example, the ramp method uses a comparator with a constant threshold and a second one with a dynamic threshold voltage (a ramp). When the output of the amplifier exceeds the constant threshold, the timestamp is recorded, and the dynamic threshold is triggered. Once the dynamic threshold is crossed down, a second timestamp is stored to obtain a more accurate ToT and a measurement of the maximum amplitude of the amplified signal. This method has been applied to MuPix8, achieving a TW of around 14 ns before correction [11] and 6 ns after the offline ToT correction [13].

Another approach is the so-called analog sampling method, which consists of sampling the leading edge of the amplified signal and fitting the linear response to the obtained data points. The intersection between the fitted leading edge and the baseline voltage gives the time of arrival with high accuracy. The main drawback of this method is that either the analysis of the points must be implemented on the sensor, where the area and power consumption are extremely limited or more data must be read out per hit. In LF-ATLASPix [14], this method was implemented using sample and hold and several capacitors as analog memories, each of them retaining the charge corresponding to a sample of the amplified signal. Then, using a single-ramp ADC converter, a current was injected into each capacitor, and the discharge times were stored as time stamps. Measurements showed a TW of 10 ns using six sampled voltage points at the cost of reading out 48 additional bits.

In Ref. [15], using the MPW2 readout circuitry of the RD50 collaboration, analog sampling of the rising edge of the amplified signal is achieved by adding additional lines (5). The first line is used to detect the rising edge and to generate five additional delayed signals. These other signals control the sampling (five points) and storage of the preamplifier output voltage value into a capacitor, while the output of the discriminator activates the storage of the TS. Additionally, as the sampling method allows for achieving time resolutions as low as the time sampling window, thus, a five-stage time-to-digital converter (TDC) using an emulated 80 MHz master clock is also added to reduce the bin to 2.08 ns. The 62 µm × 62 µm pixel simulations predict a corrected TW of 2.08 ns for energies from 1000 e^−^ to 10,000 e^−^ at a cost of five additional lines, an increase in the power consumption of 28 µW (100% of the power consumption of the RD50 current solution), and a demanding off-pixel ADC (which requires high speed and resolution) to convert the sampled data.

To the authors’ knowledge, the best corrected TW is the one obtained using the hybrid pixel detector TimePix4 [16]. Not being a DMAPS, TimePix4 does not have such stringent limitations on available area in the pixel, and the readout electronics can benefit from the technology downscaling in terms of power consumption and speed. However, as mentioned above, it establishes the state of the art and, therefore, it is interesting to analyze this approach.

In Timepix4, the 50 µm × 50 µm pixels in a double column are grouped in super-pixels (2 × 4). Each super-pixel shares a voltage-controlled oscillator (VCO), which provides a 640 MHz reference clock to generate a highly accurate in-pixel TS. Four versions equally shifted in phase of this fast clock are also generated. By registering the state of all four clocks, a TDC bin of 195 ps is achieved. The proposed solution simultaneously registers the measured ToA and ToT for energies between 800 e^−^ and 8000 e^−^ using 47 bits. According to the reported experimental results, the resolution of 195 ps is only achieved for energies higher than 7000 e^−^, and for a minimum energy of 2000 e^−^, it is slightly below 5 ns [16,17]. The power consumption of the solution is kept below 1 W/cm^2^ thanks to the use of scaled standard CMOS technology.

From the previous analysis, it can be concluded that the most promising solutions are those based on TDCs, which also perform off-pixel correction based on a highly accurate ToT. Therefore, the aim of this work is to propose a highly efficient timing solution based on TDCs. To comply with this objective, we develop a methodology to optimize the bin size of the required TDCs according to the target time resolution, exploiting the asymmetry of the CSA output. Additionally, we propose an efficient implementation of distributed TDC, giving heedful attention to considerations at both the pixel and matrix levels.

## 3. RD50-MPW3 Solution

**Figure 3:**
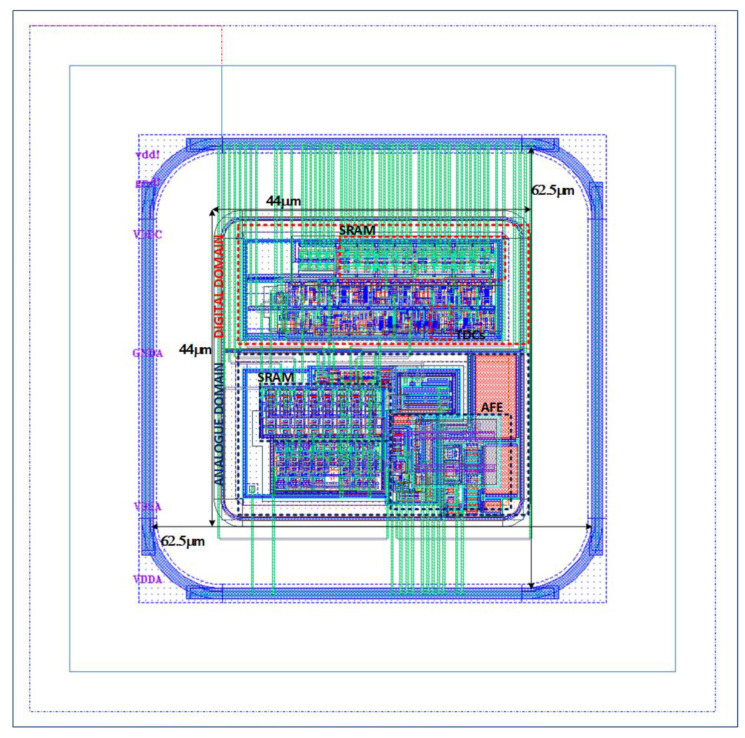
RD50-MPW3 Pixel.

The starting point of this work is the RD50-MPW3 [18], which is a two-column-based matrix of 64 × 64 pixels. Each pixel consists of a 62 µm × 62 µm size sensing diode that integrates both the analog and digital readout electronics in an area of less than 44 µm × 44 µm (Figure 3).

**Figure 4:**
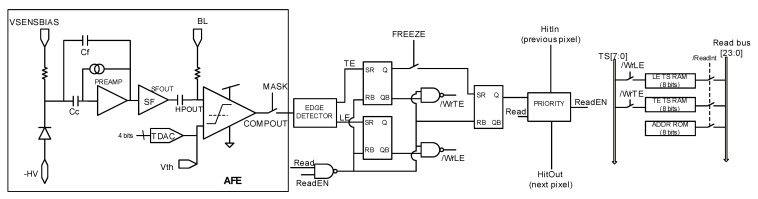
RD50-MPW3 pixel readout electronics.

The analog front-end (AFE), inherited from RD50-MPW2 [19], is shown in Figure 4 and consists of the biasing circuit required for the sensing diode, a preamplifier, a high-pass filter that also sets the signal at a base line (BL) voltage, and a comparator whose threshold level can be fine-tuned using an in-pixel 4-bit trim-DAC. The overall quiescent power consumption of the RD50-MPW3 is 28 µW.

**Figure 5:**
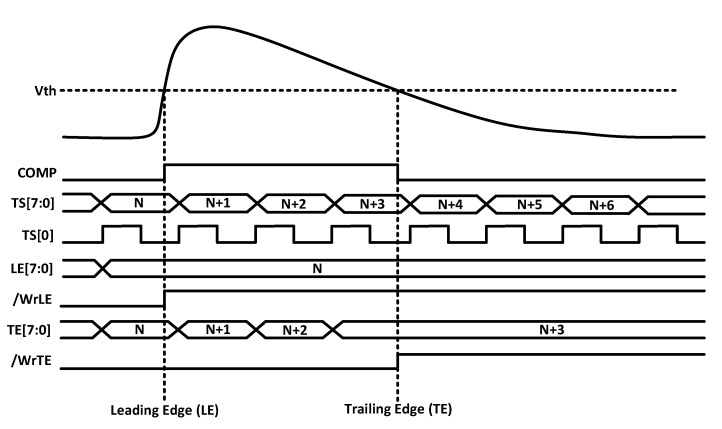
Time diagram of the RD50-MPW3 in-pixel acquisition logic.

The output of the comparator is processed by the digital readout. This circuitry includes an 8-bit ROM to store the pixel address within the double column and two 8-bit RAMs that store the TS of the leading edge (TS_LE_) and trailing edge (TS_TE_). Specifically, an 8-bit gray-encoded TS running at 40 MHz is continuously written in the RAMs. Once a hit event is detected, the last stored TS is held, and the HitOut flag is set, disabling the processing of a new event until the pixel is read. The HitOut flag is also sent to the following pixels. Figure 5 depicts the time diagram of the digital logic described above. Note that the read of the pixel after a hit is based on a priority OR chain.

The main limitation of the RD50-MPW3 proposal, in terms of resolution, is that the TS_LE_ cannot be measured with an accuracy better than 25 ns, which corresponds to the TS generated from the 40 MHz master clock (T_CLK_). This work aims to improve the time resolution of the detector to a few nanoseconds—established by the SoA for DMAPSs at 2.08 ns—by correcting the TW using a highly accurate measurement of the ToT while guaranteeing low power consumption, small area for the in-pixel electronics, and compatibility with the RD50-MPW3 solution. Table 1 summarizes the requirements derived from the RD50 solution.

## 4. Theoretical Analysis for Architecture Optimization

This section theoretically analyzes the implications of using a TDC-based timing solution to perform ToT correction, the potential of which is clearly identified in Section 2 and proposes a methodology to optimize its architecture according to the targeted resolution and TS precision.

According to (1), ToT is defined as the time difference between the TS_LE_ and TS_TE_ events. Therefore, to increase the accuracy of ToT acquisition, two additional TDCs will be required, one for each event. These two TDCs will be added to the current flip flops that store the TS_LE_ and TS_TE_ in the RD50-MPW3 solution to build a coarse–fine TDC. Note that we will focus on delay-line-based TDCs, as they have demonstrated to be area efficient and robust after fabrication [20].

The time resolution when using delay-line-based TDCs is given by

(2) $$T_{\mathrm{bin}}=\frac{{\mathrm{TS}}_{\mathrm{clk}}}{N_{\mathrm{bins}}}=\frac{{\mathrm{TS}}_{\mathrm{clk}}}{N_{\mathrm{delays}}+1}$$

where T_bin_ is the size of the bin, TS_CLK_ is the period of the clock used to generate the time stamp, N_bins_ is the number of bins, and N_delays_ is the number of delay stages of the voltage-controlled delay line (VCDL).

**Figure 6:**
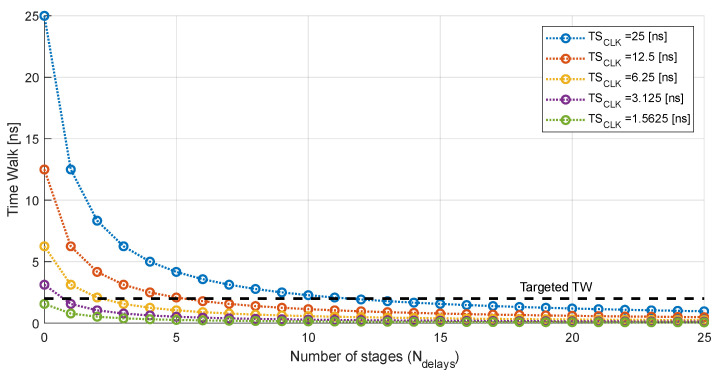
Reduction in the time walk according to the number of stages that integrate the TDC.

In the case of the RD50-MPW3, the master clock runs at 40 MHz. This allows for the estimation of the TS_LE_ with an accuracy of 25 ns after correction with the ToT. References [15,16] proposed to measure the ToT with the precision desired for ToA acquisition. Therefore, to achieve a resolution of 2.08 ns with a master clock of 40 MHz, 11 delay stages are required in each of the TDCs (see Figure 6). This implies including, in each pixel, 22 D flip flops and 11 delay stages in the VCDL, which means an unacceptable increase in the area and power consumption. Note that delay lines can be designed to perform zero quiescent power consumption; however, they can be significantly power hungry during normal operation, depending on the implementation of the TDC.

First, an analysis is performed on the impact of the frequency of the master clock, and then a methodology based on the asymmetrical shape of the leading and trailing edge of the amplified signal (Sfout in Figure 4) is proposed to reduce the required number of stages of the TDCs.

### 4.1. Increasing the Frequency of the Master Clock

According to (2), the most straightforward approach to reduce the number of required stages is to increase the frequency of the master clock. However, as mentioned in the Introduction, issues arise with a drastic increase in the frequency. First, the complexity of the layout escalates due to new critical constraints to satisfy the signal integrity in the whole matrix. Additionally, the switching noise will grow, degrading the sensitivity of each pixel. Lastly, the total dynamic power consumption will rise considerably. Therefore, there is a compromise between the required electronics (minimum number of cells that fit in the pixel) and the operating frequency. In this work, special attention was paid to the remaining area of the 44 µm × 44 µm island from the RD50-MPW3 pixel, and an 80 MHz master clock was selected. In this scenario, although the dynamic power consumption will be higher, the required area is significantly reduced as the number of delay stages of each TDC is reduced, according to Figure 6, to five (six bins). Furthermore, no significant signal integrity or noise issues are envisioned. In any case, the necessary layout considerations will be taken to minimize the impact of increasing the clock frequency.

### 4.2. Optimizing the Number of Stages in the TDC

**Figure 7:**
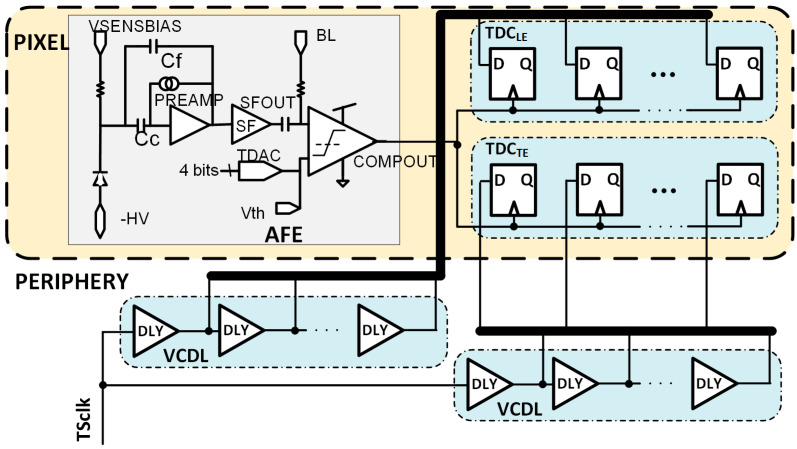
Block diagram of the modified RD50-MPW3 pixel architecture.

Figure 7 depicts a modified architecture of the RD50-MPW3 pixel, including the two TDCs. TDC_LE_ will provide a fine measurement of the TS_LE_ and TDC_TE_ of the TS_TE_.

**Figure 8:**
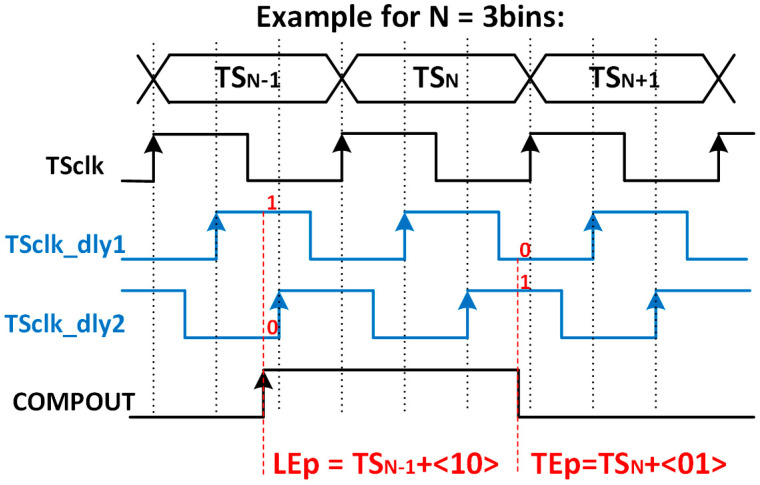
Time diagram of the TDC-based solution.

Their principle of operation is shown in Figure 8, where the number of bins is limited to three for simplicity. The VCDL acts as a phase generator, since the output of each stage corresponds to a delayed version of the clock used to generate the TS. When the amplified signal surpasses the discriminator threshold, a leading-edge event occurs, the output of the AFE (COMPOUT) is asserted, and, consequently, the TDC_LE_ is triggered. At that moment, the TS and the VCDL output are stored. Therefore, the leading edge is detected, in this example, at $TS_{LE}=TS_{N-1}+1\cdot T_{bin}$, which corresponds to the second bin of the TS_N−1_. Similarly, when the amplified signal crosses down the discriminator threshold, a trailing-edge event occurs, and COMPOUT is deasserted. Consequently, the TDC_TE_ is triggered, storing the TS and the output of the VCDL. Thus, the trailing edge is detected, in this example, at $TS_{TE}=TS_{N}+2\cdot T_{bin}$. Note that both TDCs do not replace but complement the existing TS registers depicted in the original pixel (Figure 4), now becoming coarse–fine TDCs.

To optimize the number of stages in each of the required TDCs, a high-level model of the proposed system was developed. This model is divided into three blocks: the AFE response emulator, the TW estimator, and the TW corrector.

**Figure 9:**
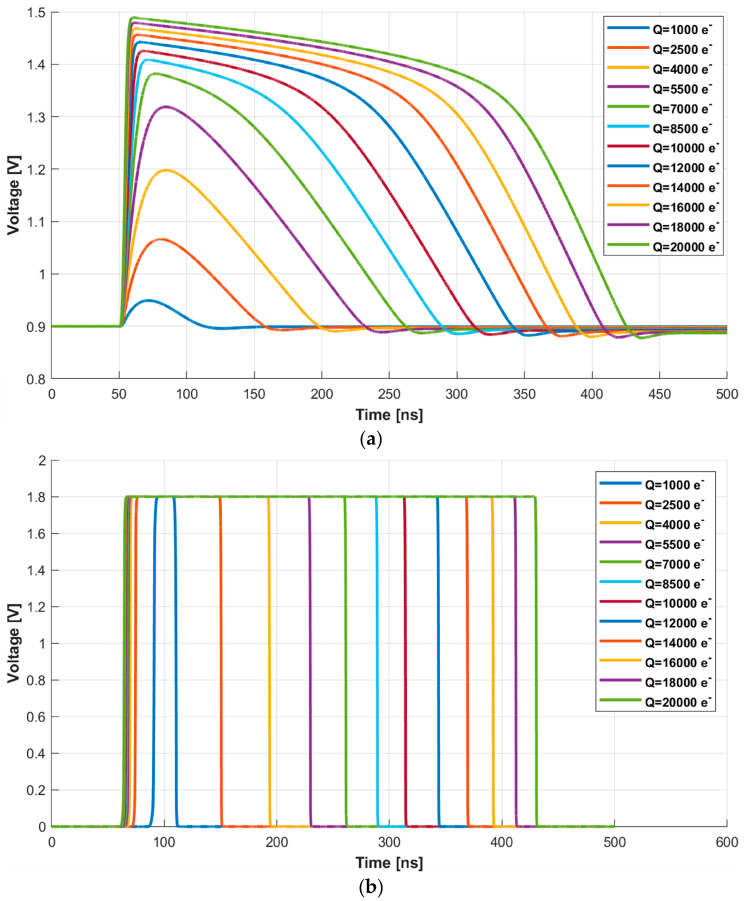
(**a**) Waveforms at HPOUT and (**b**) COMPOUT for energies from 1000 e^−^ to 20,000 e^−^ in 1500 e^−^ and 2000 e^−^ steps.

The first block receives, as input, post-layout simulations of the MPW3-RD50 AFE and randomizes the impact time of the energy particles. Specifically, the post-layout simulations are performed for energies in a range from 1000 e^−^ to 20,000 e^−^ with a total number of 70 points uniformly distributed. Figure 9a shows the response of the source follower after the preamplifier (HPOUT in Figure 4), while Figure 9b depicts the output of the comparator (COMPOUT). Note that, for clarification purposes, the figure shows curves spaced in 1500 e^−^ energy steps for impacts below 10,000 e^−^ and 2000 e^−^ steps for the 10,000 e^−^ to 20,000 e^−^ range.

Regarding the impact time, it is considered that an impact can occur, from a certain time instant, t_0_, at any moment in a time window equal to two TS_CLK_ without a lack of generality. Consequently, a new set of waveforms (based on post-layout simulations) with randomly generated impact time is created.

**Figure 10:**
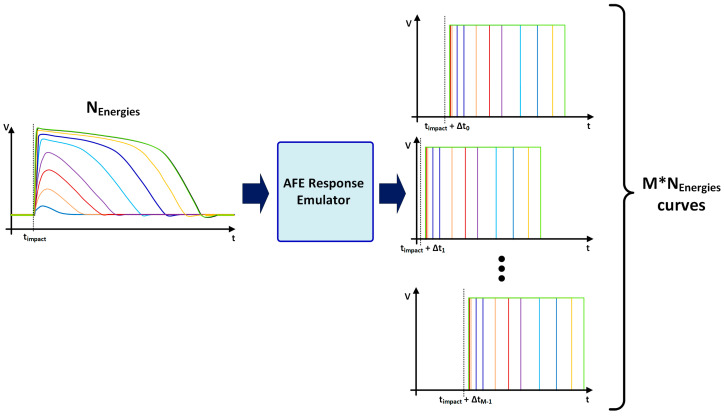
Curves generated by the AFE response emulator.

Then, each of these sets of curves (Figure 10) is used, in the second block, to determine the TS_LE_ to be recorded by the TDC_LE_. Similarly, the instant at which the trailing edge is detected will be used to determine the TS_TE_ to be stored by the TDC_TE_.

Therefore, this first block is responsible for generating M*N_energy_ curves, where N_energy_ = 70 represents the number of simulated energy levels and M = 50,000 is the number of simulated impacts with different impact times for the same energy. Note that the impact time of each set of curves is uniformly distributed between t_0_ and t_0_ + 2 TS_CLK_ and the total number of curves is then 3,500,000, which constitutes a statistical population that ensures consistent results for the following analysis.

After the different curves have been generated, the second block oversees discretizing the time instants of the leading (TS_LE_) and trailing (TS_TE_) edges of the output of the comparator for different combinations of the number of stages for the TDC_LE_ and TDC_TE_. These numbers will be represented by N_LE_ and N_TE_, respectively.

Finally, the third and last block computes, from the same family of curves, the difference between the measured TS_LE_ for each energy with respect to TS_LE_ of the maximum energy, $TS_{LE}^{max,Ener}$.

This cost function ε is given by

(3) $$\varepsilon ={\mathrm{TS}}_{\mathrm{LE}}-{\mathrm{TS}}_{\mathrm{LE}}^{\max,\mathrm{Ener}}$$

Regarding the correction, it is based on an LUT that registers the acquired ToT, the TS_LE_, and the computed ε for each hit of the considered statistical population. It consists of subtracting the computed ε from each stored TS_LE_. Note that in some cases (particularly in those with low hit energies), different values of ε can be associated with the same acquired ToT. In those cases, the correction factor assigned to that ToT is set to the minimum of all the associated ε. Those cases will lead to the least accurate results and will set the final time resolution after correction.

**Figure 11:**
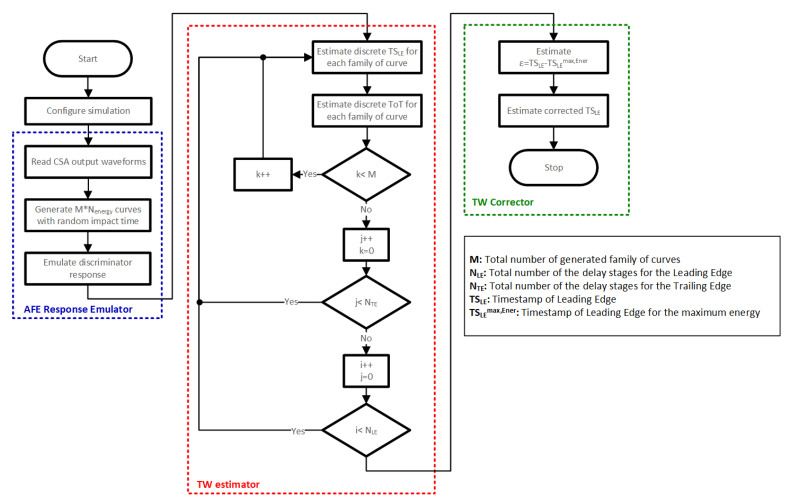
Flow chart of the system high-level model.

Figure 11 depicts a flow chart corresponding to the above-described system high-level model.

**Figure 12:**
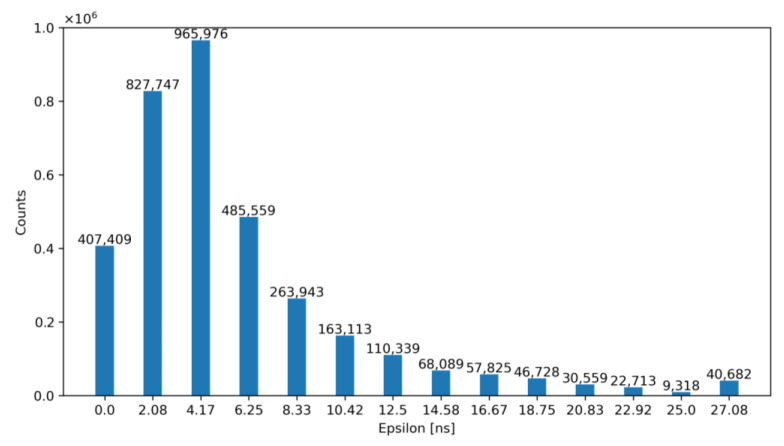
Histogram of the obtained ε for N_LE_ = 5 and N_TE_ = 5 before correction.

**Figure 13:**
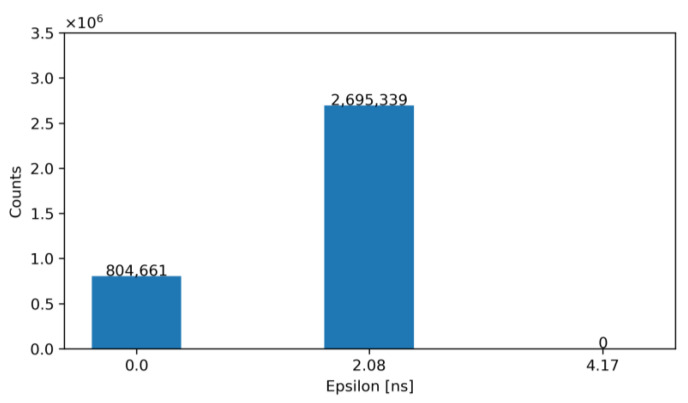
Histogram of the obtained ε for N_LE_ = 5 and N_TE_ = 5 after correction.

For exemplification purposes, Figure 12 and Figure 13 show the output of our model—before and after correction—for an architecture with N_LE_ = 5 and N_TE_ = 5 for TDC_LE_ and TDC_TE_, respectively. Specifically, Figure 12 shows the number of occurrences of the obtained ε before correction, while Figure 13 illustrates the same error after correction with the ToT. Note that, according to these figures, the TW before correction is 27.08 ns and after correction is equal to 2.08 ns.

**Figure 14:**
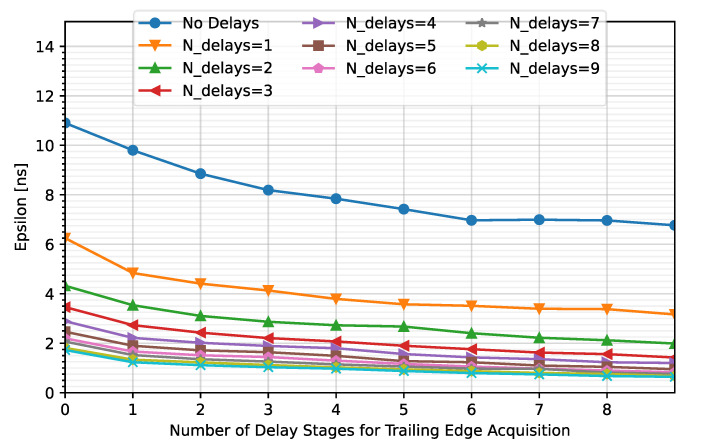
Variation in the meantime error for different number of stages for TDC_LE_ and TDC_TE_.

To optimize the number of stages in each TDC (TDC_LE_ and TDC_TE_), simulations were performed considering up to nine stages for each of the TDCs. Figure 14 shows the average value of ε after correction against the number of stages that compose the TDC_TE_. Note that each curve represents a different N_LE_. Additionally, cases where the comparator output is not captured with a TDC converter (N_LE_ = N_TE_ = 0) were included to determine the impact on the error.

**Figure 15:**
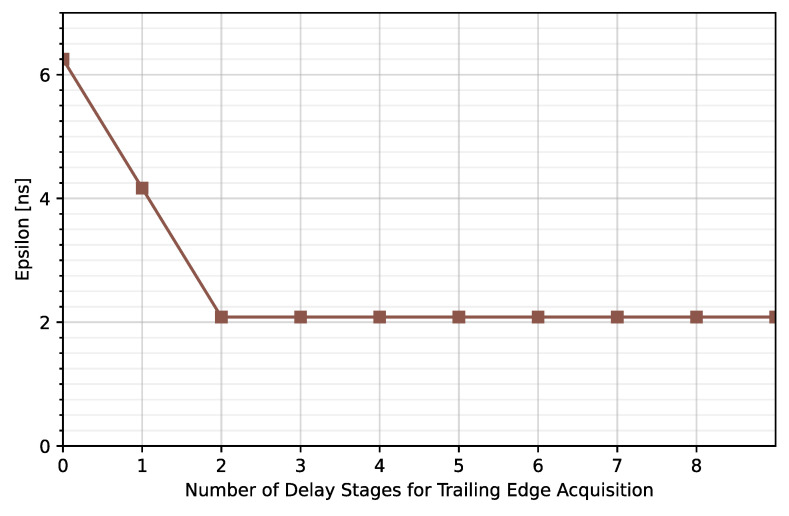
Maximum time error for N_LE_ = 5 and different N_TE_.

As can be observed, the higher the number of stages composing the TDCs, the lower the timing error. Note that an average error of less than 2.08 ns is achieved for N_LE_ = 5 and N_TE_ ≥ 1 but the maximum error is kept under 2.08 ns for N_LE_ = 5 and N_TE_ ≥ 2 (Figure 15). Therefore, N_TE_ = 2 instead of N_TE_ = 5 will be considered for this solution.

## 5. Proposed Implementation

After optimizing the number of delay stages for each TDC, the practical implementation is addressed in this section. Several alternatives have been proposed, studied, and successively refined to find an efficient implementation that meets the system requirements (detailed in Table 2).

**Figure 16:**
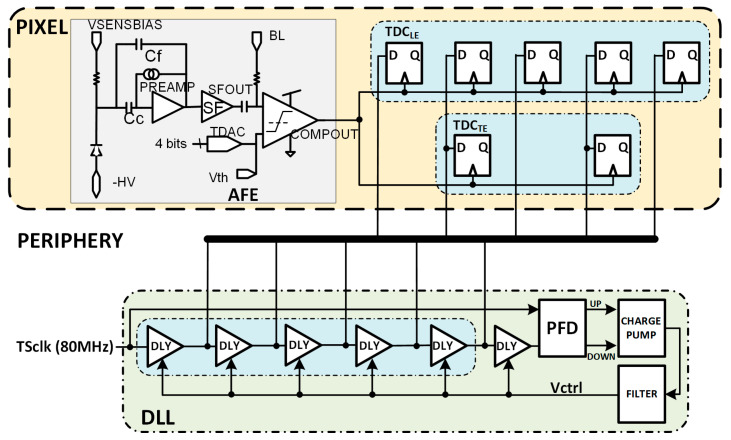
Pixel architecture including the DLL in the periphery.

It is important to highlight that, as in the case of N_LE_ = N_TE_ = 5, the combination of N_LE_ = 5 and N_TE_ = 2 avoids the need for two VCDLs, one for each TDC. A delay line with five stages fits the requirements of the TDC_LE,_ and the output of the second and fourth stages can be reused in the TDC_TE_ (see Figure 16).

The VCDL generates the five clock phases that must be controlled by a delay-locked loop (DLL) to cope with intrinsic process variability and compensate for ageing and radiation effects [21]. The DLL is fed with the clock used to generate the TS, adjusting the delay of each VCDL element to a sixth part of the clock period and generating six bins of equal size. The most convenient approach seems to be to locate the DLL in the periphery of the matrix, shared by all pixels in a column (Figure 16). This avoids significant dynamic power consumption and helps with the area limitations inside the pixel. The main drawback of this solution is to route five high-frequency clock phases along the pixel column. This implementation would consume significant routing resources, limiting either the minimum size of the pixel or the minimum distance between them and, therefore, the spatial resolution that can be achieved. This complex routing can also jeopardize the signal integrity of the rest of the chip, which is critical, as the accuracy of the time-to-digital conversion strongly depends on the precision of these five clock phases.

**Figure 17:**
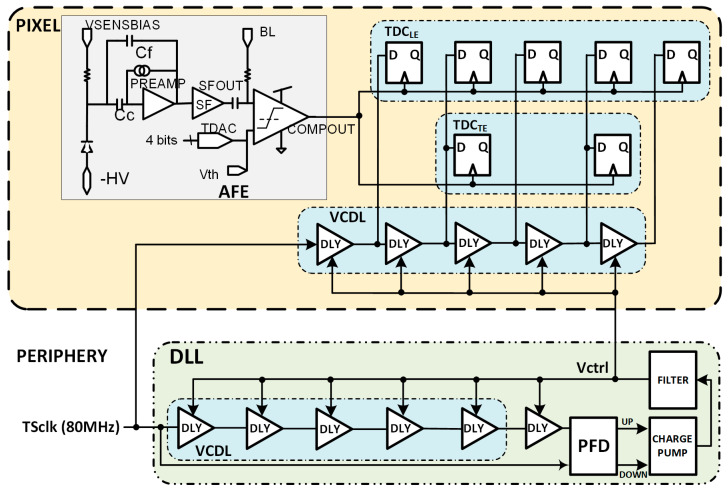
Pixel architecture including a replica of the VCDL.

An alternative to the previous is to integrate a replica of the VCDL in each pixel. This solves the routing problem at the cost of increasing the pixel area and dynamic power consumption. As shown in Figure 17, the DLL in the periphery adjusts the delay of each cell to a sixth part of the TS clock period by locally controlling the Vctrl voltage. Then, the Vctrl signal is routed to each pixel. In this solution, only two lines should be routed from the periphery to each pixel (Vctrl and TS_CLK_), drastically decreasing the required routing resources. Another advantage of this architecture is that clock phases are generated locally in the pixel, helping to maintain the integrity of the signal and, consequently, the timing accuracy. On the other hand, this solution leads to a substantial area of the electronics inside the pixel and an unsustainable increase in power consumption of the in-pixel circuitry due to a high-frequency clock signal continuously running through the VCDL.

**Figure 18:**
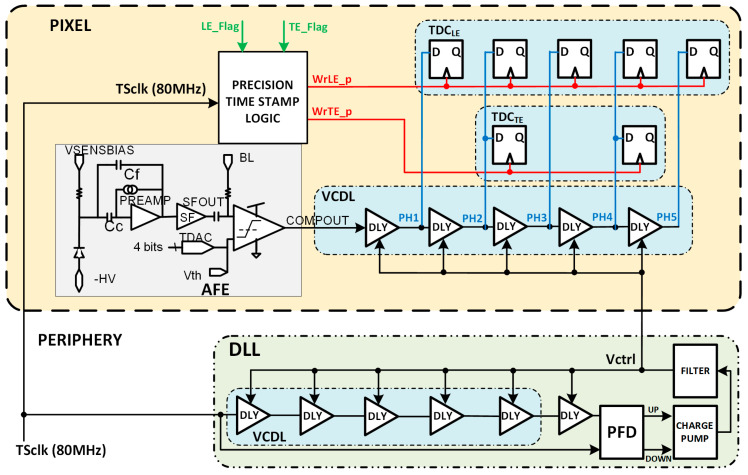
Pixel architecture with COMPOUT connected at the input of the VCDL.

To avoid this problem, the output of the comparator (COMPOUT) can be connected to the input of the VCDL instead of the TS_CLK_ (Figure 18). By doing this, the in-pixel VCDL generates five versions of COMPOUT delayed 2.08 ns instead of five delayed phases of TS_CLK_. Therefore, the in-pixel VCDL cells are commuted only twice per hit, significantly decreasing the dynamic power consumption compared to the previous implementation. As COMPOUT is no longer used as the stop signal for the TDCs, in this implementation, additional logic (precision time stamp logic) is needed to generate new stop signals, WrLE_p and WrTE_p, on the next clock edge to the LE and TE events.

**Figure 19:**
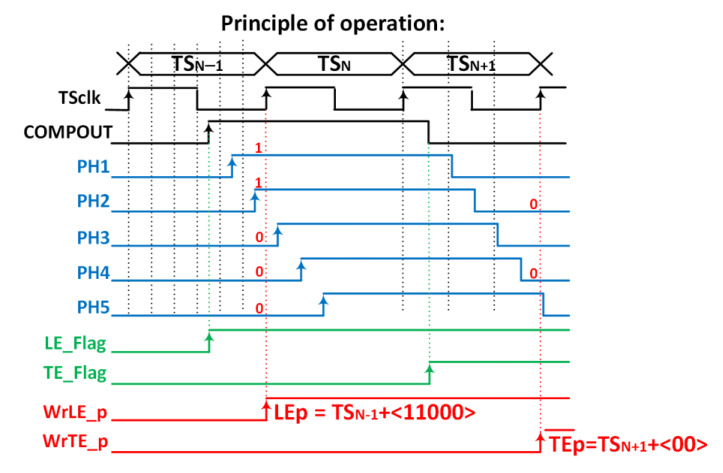
Principle of operation.

The chronogram in Figure 19 shows the principle of operation using a hit with LE and TE in the fourth and first bins of each corresponding TS. The time delay of each cell in the in-pixel VCDL is controlled by the DLL in the periphery. The in-pixel VDCL outputs (labelled as PHx) are the five phases of COMPOUT delayed by 2.08 ns. In the case of an LE event, the five phases are captured on the first rising edge of TS_CLK_ after COMPOUT is asserted, measuring the time between the LE event and the end of its corresponding TS. The five phases are stored in the corresponding TDC_LE_ register. When read out, the stored code provides enough information to identify the time bin when the event happened: the more ‘1′s in the code, the earlier the LE event occurred. The procedure in the TE event is similar, but only phases two and four are stored, dividing the corresponding TS into three bins. In this topology, the in-pixel VCDL consumes only when a particle hits the pixel. After that, power consumption is negligible until the pixel is read out. However, additional logic is required to generate WrLE_p and WrTE_p signals, which must be asserted to store LEp and TEp codes. This slightly increases the in-pixel area occupied by the new timing circuitry.

**Figure 20:**
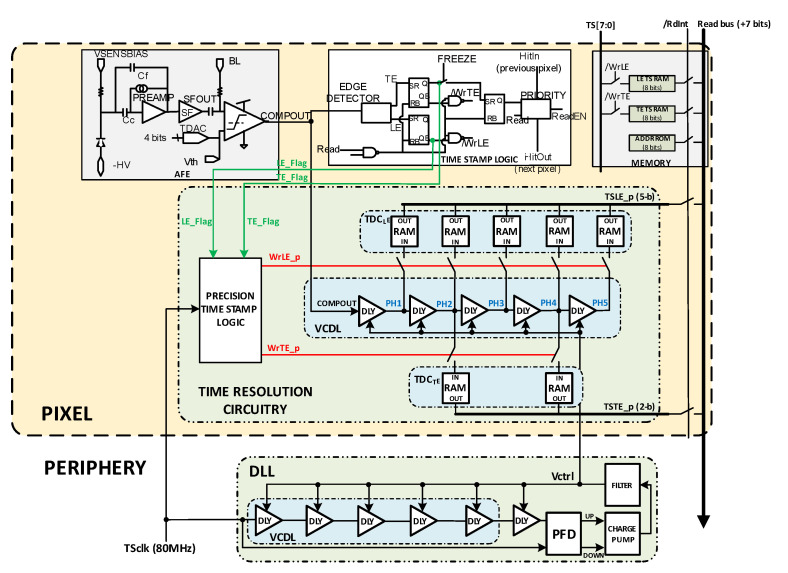
Final implementation.

Figure 20 shows the final proposed implementation. The signals LE_Flag and TE_Flag are determined by the readout logic already present on the RD53-MPW3 solution when the TS corresponding to LE and TE is stored. These signals allow the new circuitry to generate WrLE_p and WrTE_p signals with the next TS_CLK_ rising edge performing the storage of the VCDL output. In the final implementation, D-RAM cells were used to build the TDC registers instead of D flip flops, as they are more efficient in area and better fit to the RD50-MPW3 chip implementation. The readout flow and priority methodology of the RD50-MPW3 chip were also maintained to ensure compatibility. For a double-column read-out, only the pixel with active priority will force a ‘1′ in the /RdInt signal. Then, information stored in the DRAM registers is written in the readout bus, shared by all the double-column pixels.

## 6. Simulation Results

In this section, the simulation results for the proposed architecture are shown. Standard digital cells and classic topologies were used for implementation to evaluate the viability of the solution.

**Figure 21:**
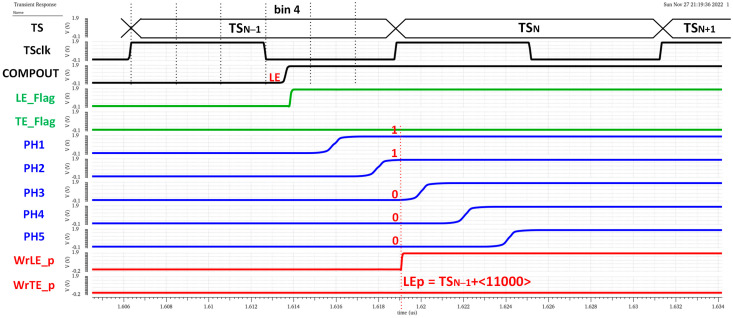
LE time measurement.

Figure 21 shows a time diagram of the simulated pixel when detecting an LE event in bin 4 of TS_N−1_. In the readout process, the estimated T_LE_ has an accuracy equal to 2.08 ns (T_bin_) and is given by

(4) $${\mathrm{LE}}_{p}={\mathrm{TS}}_{N-1}+“11000”$$

**Figure 22:**
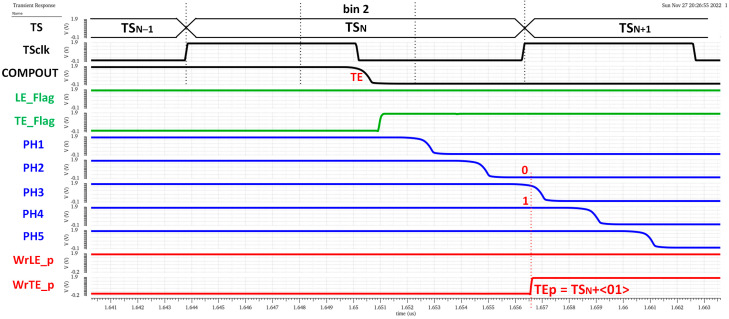
TE time measurement.

Note that the complete LE time measurement is then composed of two parts, coarse time information given by TS_CLK_ (registered by the original TS logic circuitry) and fine time information given by the new timing circuitry. The higher the number of ‘1’ in the LE_p code, the earlier the LE event occurred during the corresponding TS_CLK_. Similarly, Figure 22 shows a time diagram of the simulated pixel when detecting a TE event in bin 2 of TS_N_. In this case, the fine time information of a T_TE_ estimation is taken only from phases two and four, dividing the TS into three equal parts and providing a 4.17 ns accuracy in T_TE_. In this case, the polarity is complementary, so the more ‘0’ in the TE_p code, the earlier the TE event occurred during the registered TS_CLK_.

**Figure 23:**
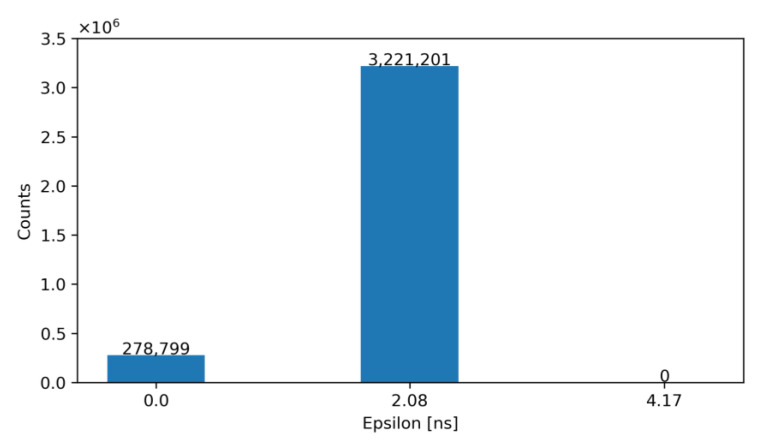
Histogram of the obtained ε for N_LE_ = 5 and N_TE_ = 2 after correction (proposed solution).

Table 3 summarizes the results obtained from the previous simulations. To make a comparison, the TW achieved (Figure 23), as well as the estimated area and power consumption for the two architectures discussed, is gathered. Additionally, the table collects if the implemented topology requires additional input/output terminals, which is relevant for the physical implementation in the pixel matrix.

According to the data presented, architecture 2 (N_LE_ = 5 and N_TE_ = 2) is the most efficient in terms of both implemented area and routing complexity. Specifically, it reduces from 10 to 7 the required outputs for the timing readout of LE and TE compared to the classical approach and reduces the area by 15% with respect to architecture 1 (based on the area of the available standard cells). The area of the proposed solution is estimated at 279 µm^2^, which fits the remaining area of 319 µm^2^ available for electronics in the RD50-MPW3 pixel.

It is important to highlight that due to the stringent limitations of space inside the pixel, any reduction in the area required by the timing electronics is valuable. Furthermore, when targeting higher resolutions, the impact of using the proposed methodology is even more significant. For example, for TW = 1.04 ns, our model recommends using N_LE_ = 10 ns and N_TE_ = 4 (versus N_LE_ = 10 ns and N_TE_ = 10) without degradation in time resolution. The estimated area for the physical implementation is expected to be reduced by 20%.

Simulations show that after off-pixel correction with the measured ToT, the average time resolution of the ToA is 1.71 ns and the system TW is 2.08 ns for energies in a range from 1000 e^−^ to 20,000 e^−^.

Table 4 compares the performance of the proposed solution with the most relevant proposals from the SoA (Section 2). Works under consideration aim at pixel detectors with improved time resolution. The pixel size in references [9,14] prevents one from using them in systems that require high granularity. In addition, ref. [9] does not integrate the time acquisition circuitry, and time measurements are made outside the chip using an oscilloscope.

Solutions that locate the time acquisition electronics in the periphery, such as MuPix (refs. [11,12,13]), generally do not achieve the best time resolution. On those including the timing electronics (or part of it) in the pixel, the resolution is better. The authors of [15] achieved high resolution for high power ranges (>6000 e^−^) but at the cost of doubling the in-pixel power consumption and needing to include a high-performance ADC in the periphery.

Finally, [16] sets the state of the art to 195 ps for energies higher than 7000 e^−^. However, it is a hybrid solution that takes advantage of designing the readout electronics in a 65 nm CMOS technology, and the resolution degrades to around 5 ns for a minimum energy of 2000 e^−^. Moreover, it requires 48 extra lines. Note that our proposal outperforms the comparison in terms of time resolution for monolithic solutions in which high granularity is required. It achieves a 2.08 ns resolution for energies between 1000 e^−^ and 20,000 e^−^ at zero additional quiescent power consumption, and it requires only 23 additional lines.

## 7. Conclusions

DMAPSs are an interesting choice for future HEP experiments because of their low cost and high robustness to radiation. However, the requirement of high spatial resolution imposes stringent area and power consumption specifications on the in-pixel readout electronics, limiting the system performance and, thus, leading to poor time resolution.

Several approaches have been proposed to improve the time accuracy in DMAPSs. The most common solutions are based either on analog-to-digital conversion or time-to-digital conversion. Analog-based solutions are generally area-efficient; however, the high hit rate of these systems implies a high-speed ADC and, thus, high complexity, large area, and excessive power consumption. Locating the timing electronics in the periphery relaxes this problem, but the resolution deteriorates due to the dispersion in the delays of the signals that arrive at the pixel, depending on its position within the matrix. Digital-based solutions use a TDC to accurately measure the ToA and, when combined with offline correction using the ToT, they have been demonstrated to offer the best time resolution.

This work proposes an area- and power-efficient solution that can be fully integrated with the implementation of RD50-MPW3. It is based on two TDCs of minimal complexity included in each pixel. To optimize their architecture, a methodology was proposed to determine the maximum bin size required for each of the TDCs exploiting the asymmetry of the AFE output signal. From this study, it is concluded that the number of stages of the TDC_TE_ can be reduced by half, minimizing the required area, without compromising the resolution.

Additionally, the architecture of the TDC was rethought to avoid routing multiple clock signals from the periphery to the pixel, thus eliminating undesired propagation delays. Unlike solutions that use the master clock as the input of the VCDL, our proposal achieves zero additional quiescent consumption in the pixel and reduced dynamic power consumption because the TDC only commutes when a hit is detected.

The proposed timing solution fits into the available space for electronics in the RD50-MPW3 pixel and performs, according to simulations, a timing resolution of 2.08 ns for energies in a range from 1000 e^−^ to 20,000 e^−^. To the authors’ knowledge, the proposed solution achieves the best timing resolution published to date with no additional quiescent power consumption in the pixel.

Future research includes designing and fabricating a small-array prototype using the proposed architecture and obtaining experimental measurements to validate the solution and the achieved time resolution. Additionally, it is essential to analyze the possible variation in the time resolution across the arrays. This comprehensive analysis will provide valuable information on the performance and limitations of the system, paving the way for further improvements and/or optimizations to ensure a successful scaling to larger arrays.

## Figures and Tables

**Table 1:** RD50 requirements.

Specification	Value
Maximum in-pixel area occupancy	Minimum *
In-pixel I/O additional terminals	Minimum
Power density	<250 mW/cm^2^

* <319 µm^2^ which is the remaining area in the RD50-MPW3 pixel.

**Table 2:** System specifications.

Specification	Value
Time resolution after correction	2.08 ns
Reference clock frequency	80 MHz (12.5 ns period)
N_LE_	5 (2.08 ns bin size)
N_TE_	2 (4.17 ns bin size)
In pixel power consumption	Minimum
Maximum in-pixel area occupancy	Minimum (max. area 319 µm^2^)
In pixel I/O additional terminals	Minimum

**Table 3:** Required area and routing constraints for the different architecture options.

	Architecture 1	Architecture 2
N_LE_ acquisition	5	5
N_TE_ acquisition	5	2
Estimated Area [µm^2^]	331.5	279
Inputs/outputs required	1/10	1/7
ε_max_ [ns]	2.08	2.08
Average ε [ns]	1.28	1.71
Additional quiescent power consumption [µW]	0	0

**Table 4:** Comparison table of the performance of the proposed solution with the SoA.

	CAcTμS [9]	MuPix8 [11,12,13]	ATLASPix [14]	O. Alonso et al. [15]	Timepix4 [16,17]	This Work
Type of solution	Monolithic	Monolithic	Monolithic	Monolithic	Hybrid	Monolithic
Manufacturing technology	LF150 nm	AMS aH18	LF150 nm	LF150 nm	CMOS 65 nm (Readout)	LF150 nm
Pixel size	1 mm^2^/0.5 mm^2^	80 μm × 81 μm	150 μm × 50 μm	60 μm × 60 μm	55 μm × 55 μm	62 μm × 62 μm
Location of the timing acquisition	Off-chip	Off-pixel	In-pixel	In-pixel	In-pixel	In-pixel
Technique	Increase speed amplifier	TWCC Two thresholds Ramp	Analog Sampling + Ramp-ADC	Analog Sampling + TDC (VCDL based)	TDC (VCO based)	TDC (VCDL based)
Correction	Offline with ToT (Measured off-chip)	Offline with discriminator delay + 6-bit ToT	Offline with sampled amplitude (48 bits for sampled amplitudes + 16-bit TS)	Offline with sampled amplitude + ToT (5 analog lines + 26 bits ToT)	Offline with ToT (45 bits)	Offline with ToT (23 bits)
Additional pixel quiescent power consumption	N/A	-	-	28 μW	-	None
Total quiescent power consumption per pixel	1.44 mW	-	-	56 μW	-	28 μW
Additional elements at the periphery	N/A	Discriminator	-	ADC and Phase Generator	VCO (for each 2 × 4 pixels)	DLL
Energy range	4k e^−^ to 40k e^−^	1k e^−^ to 10k e^−^	-	>6k e^−^	>7k e^−^	1k e^−^ to 20k e^−^
Time resolution	105 ps	6.5 ns	-	2.08 ns	195 ps	2.08 ns

## Data Availability

Data available on request.

## Abbreviations

The following abbreviations are used in this manuscript:

ADC	Analog-to-Digital Converter
AFE	Analog Front-End
ALICE	A Large Ion Collider Experiment–Heavy Ion at LHC
ATLAS	A Toroidal LHC Apparatus (LHC Collaboration)
BL	Base Line
CMS	Compact Muon Solenoid (LHC Experiment)
CSA	Charge Sensing Amplifier
DAC	Digital-to-Analog Converter
DLL	Delay-Locked Loop
DMAP	Depleted Monolithic Active Pixel Sensor
DRAM	Dynamic Random Access Memory
HEP	High-Energy Physics
LE	Leading Edge
LF-AtlasPix	LFoundry-AtlasPix
LHC	Large Hadron Collider
LHCb	Large Hadron Collider beauty
LUT	Look-Up Table
MAP	Monolithic Active Pixel Sensor
MPW2	Multi-Project Wafer 2
RAM	Random Access Memory
RD50	Recherche et Développement 50
RD50-MPW2	Recherche et Développement 50 Multi-Project Wafer 2
RD50-MPW3	Recherche et Développement 50 Multi-Project Wafer 3
ROM	Read Only Memory
SoA	State of the Art
TDC	Time-to-Digital Converter
TDC_LE_	Time-to-Digital Converter for Leading Edge
TDC_TE_	Time-to-Digital Converter for Trailing Edge
TE	Trailing Edge
ToA	Time of Arrival
ToT	Time over Threshold
TS	Time stamp
TS_LE_	Time stamp of leading edge
TS_TE_	Time stamp of trailing edge
TW	Time Walk
TWCC	Time-Walk-Compensated Comparator
VCO	Voltage-Controlled Oscillator
VCDL	Voltage-Controlled Delay Line
